# Fully Automated Support System for Diagnosis of Breast Cancer in Contrast-Enhanced Spectral Mammography Images

**DOI:** 10.3390/jcm8060891

**Published:** 2019-06-21

**Authors:** Annarita Fanizzi, Liliana Losurdo, Teresa Maria A. Basile, Roberto Bellotti, Ubaldo Bottigli, Pasquale Delogu, Domenico Diacono, Vittorio Didonna, Alfonso Fausto, Angela Lombardi, Vito Lorusso, Raffaella Massafra, Sabina Tangaro, Daniele La Forgia

**Affiliations:** 1 Dip. di Diagnosi e Terapia per Immagini, I.R.C.C.S. Istituto Tumori “Giovanni Paolo II” di Bari, 70124 Bari, Italy; annarita.fanizzi.af@gmail.com (A.F.); lilianalosurdo@gmail.com (L.L.); v.didonna@oncologico.bari.it (V.D.); massafraraffaella@gmail.com (R.M.); d.laforgia@oncologico.bari.it (D.L.F.); 2 Dip. Interateneo di Fisica “M. Merlin”, Università degli Studi di Bari “A. Moro”, 70125 Bari, Italy; teresamaria.basile@uniba.it (T.M.A.B.); roberto.bellotti@uniba.it (R.B.); 3 Dip. di Scienze Fisiche, della Terra e dell’Ambiente, Università degli Studi di Siena, 53100 Siena, Italy; ubaldo.bottigli@unisi.it (U.B.); pasquale.delogu@unisi.it (P.D.); 4 INFN—Istituto Nazionale di Fisica Nucleare, Sezione di Bari, 70125 Bari, Italy; domenico.diacono@ba.infn.it (D.D.); angela.lombardi@ba.infn.it (A.L.); 5 Dip. di Diagnostica per Immagini, Azienda Ospedaliera Universitaria Senese, 53100 Siena, Italy; afausto@sirm.org; 6 Dip. Area Medica, I.R.C.C.S. Istituto Tumori “Giovanni Paolo II” di Bari, 70124 Bari, Italy; v.lorusso@oncologico.bari.it

**Keywords:** breast cancer, contrast-enhanced spectral mammography (CESM), background parenchymal enhancement (BPE), computer-automated diagnosis (CADx), feature extraction, machine learning techniques

## Abstract

Contrast-Enhanced Spectral Mammography (CESM) is a novelty instrumentation for diagnosing of breast cancer, but it can still be considered operator dependent. In this paper, we proposed a fully automatic system as a diagnostic support tool for the clinicians. For each Region Of Interest (ROI), a features set was extracted from low-energy and recombined images by using different techniques. A Random Forest classifier was trained on a selected subset of significant features by a sequential feature selection algorithm. The proposed Computer-Automated Diagnosis system is tested on 48 ROIs extracted from 53 patients referred to Istituto Tumori “Giovanni Paolo II” of Bari (Italy) from the breast cancer screening phase between March 2017 and June 2018. The present method resulted highly performing in the prediction of benign/malignant ROIs with median values of sensitivity and specificity of 87.5% and 91.7%, respectively. The performance was high compared to the state-of-the-art, even with a moderate/marked level of parenchymal background. Our classification model outperformed the human reader, by increasing the specificity over 8%. Therefore, our system could represent a valid support tool for radiologists for interpreting CESM images, both reducing the false positive rate and limiting biopsies and surgeries.

## 1. Introduction

Among women, breast cancer is currently the most frequently diagnosed cancer and the first cause of death in the world [1]. In recent years, breast cancer mortality has decreased due to the combined effect of early diagnosis and improvement in treatment effectiveness [1,2]. The systematic use of Full-Field Digital Mammography (FFDM) on the female population since the early 2000s, the progressive technological improvement of equipment for early diagnosis, and the training of dedicated operators for the disease have allowed increasingly precocious and precise diagnoses.

The FFDM diagnostic performances are variable and dependent on some properties of the breast itself, among which the density of the mammographic pattern [3,4]: the hard reading of mammograms is often linked to the low intrinsic contrast of the glandular and fibrous tissue and to the possibility of masking lesions in the context of a particularly dense mammary structure [5,6,7]. Therefore, dense breasts constitute a mammographic vulnus and this becomes particularly important in the population screening phase, where mammography is the main or even exclusive investigation. For this reason, further radiological investigation techniques are used [8] to improve diagnostic performance in this type of breasts.

With the advent of digital mammography, in the last few years, several implementations of the same technique have been developed in order to increase its diagnostic accuracy, especially in dense breasts: among these, the Contrast-Enhanced Spectral Mammography (CESM) [9,10]. This mammographic technique is based on a dual-energy exposure after a single injection of an iodinated Contrast Medium (CM), yielding a Low- (LE) and a High-Energy (HE) image from which a ReCombined (RC) image is obtained.

The LE images obtained as part of a CESM exam can be overlapped on digital images according to modern quality standards [11,12]. The RC images exploit the principle of neo-angiogenesis characterized by the formation of small blood contiguous vessels to the neoplasia in order to provide it with nutrients sufficient for growth [13,14].

Magnetic Resonance Imaging (MRI) is also based on this phenomenon, but using gadolinium-based contrast agents. However, iodinated contrast agents have similar properties, as showed in some studies with breast Computerized Tomography (CT) [15].

Unlike CESM, the MRI technique is able to characterize in a more objective way a finding according to the signal obtained from the diffusion of water molecules. This is known as Diffusion-Weighted Magnetic Resonance Imaging (DWI or DW-MRI) and, through Regions Of Interest (ROIs) corresponding to the individual lesions, it also allows for automatically calculating the Apparent Diffusion Coefficient (ADC). In this way, it is possible to express further evaluations on the diagnosis of benignity or malignancy of a lesion. However, MRI exams are more expensive than the traditional ones [16,17,18] and are contraindicated in women who are particularly obese, poorly mobilized, suffer from claustrophobia, or have pacemakers, splinters, clips, not-titanium prostheses or other type of ferromagnetic material implanted in their body.

CESM, as well as magnetic resonance, may present different degrees of Background Parenchymal Enhancement (BPE): this represents how much the normal tissue is impregnated after the CM injection and depends on several factors, such as tissue vascularity and permeability, endogenous and exogenous hormones, and endocrine therapy effects [19,20]. The BPE degree is evaluated according to four qualitative categories: minimal, mild, moderate and marked [20,21]. Its value seems to be also correlated with the breast density and the volume of fibroglandular tissue and, according to [22], it represents a risk factor for the development of breast cancer. Since CESM is less influenced by hormonal status than MRI [23], this could provide important additional information on the detection of lesions in patients with a high (moderate or marked) degree of BPE in which it is objectively difficult to distinguish a lesion from the non-enhanced background.

Expert systems for characterizing ROIs may offer radiologists a reliable support in the evaluation of CESM images to improve accuracy of breast lesion identification in the presence of parenchymal background. Hence, the development of Computer-Automated Diagnosis (CADx) systems for breast lesions using CESM images is an important challenge.

Nevertheless, recent literature has concerned the diagnostic performance of human readers on CESM images with respect to those of mammographic and MR images. On the contrary, the state-of-the-art is poor about the development of expert systems as diagnostic support tools. In [24], the authors proposed a CADx aimed to increase the diagnostic performances of CESM compared with those obtained by experienced radiologists. This algorithm used a set of morphological and textural features extracted from low-energy and recombined images in order to train a Support Vector Machine (SVM) [25] classifier for the recognition of benign and malignant lesions. In [26], a deep learning support system is presented to improve the specificity of breast cancer diagnosis by CESM images. These expert systems provided complementary information to radiologists; nevertheless, they needed a manual segmentation of the lesion by radiologists.

To make the lesion analysis more objective and less operator-dependent, in this paper, we proposed a fully automated model which allows an efficient recognition of both benign/malignant ROIs and normal/abnormal tissues characterized by mild or high parenchymal background. From each suspicious area, manually identified by an expert radiologist, we extracted texture features in a fully automated manner without further human operation or indication about the semiotics of lesions. In order to face the fundamental challenge of improving of the breast lesion characterization, and then to decrease unnecessary biopsies and later surgeries, in the proposed model, an important role could be played by the feature extraction and selection processes used to describe and characterize ROIs.

## 2. Materials and Methods

### 2.1. Materials

#### 2.1.1. CESM Examination

**Figure 1:**
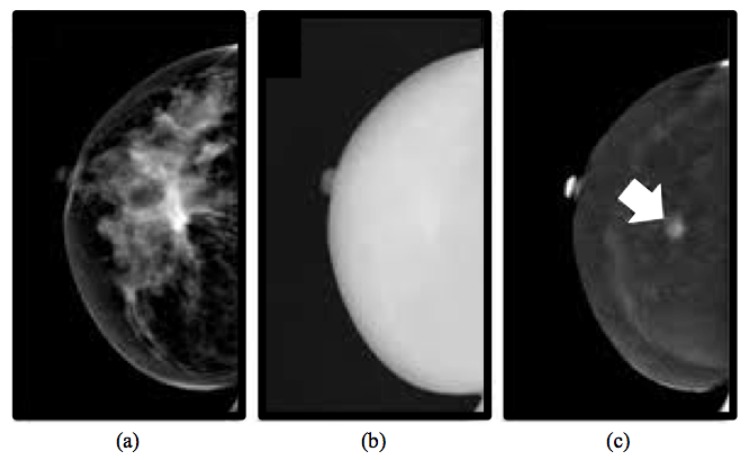
Typical example of images obtained from a CESM examination [27]: low-energy (**a**), high-energy (**b**) and recombined (**c**) images. A suspicious lesion is pointed by a white arrow on the recombined image, masked by the denser breast parenchyma on the low-energy image.

CESM is an imaging technique allowing the acquisition of multiple views of both breasts by producing two types of images: a low-energy image (Figure 1a) and a high-energy image (Figure 1b). However, the latter is not displayable in the reporting monitor, but it is necessary for the creation of a recombined image (Figure 1c) that emphasizes the breast areas with greater angiogenesis, as it occurs exactly in the breast MRI [27].

**Figure 2:**
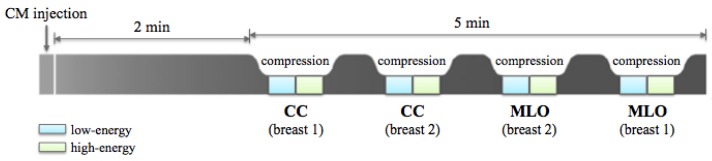
CESM examination: the diagram shows the steps of the image acquisition in the standard CranioCaudal (CC) and MedioLateral Oblique (MLO) views, after the iodinated Contrast Medium (CM) injection. Breast 1 stands for the breast with no pathology, while breast 2 is the breast with one or more lesions.

For all CESM exams, a modified FFDM system derived from a standard Senographe Essential (GE Healthcare) was used. A single-shot intravenous injection of 1.5 mL/kg of body mass of iodinated contrast agent (Visipaque 320 mg I/mL) was then performed at a flow rate of 2–3 mL/s by using an automated injector. Two minutes after the CM injection, a set of images was acquired in quick succession while the breast remains compressed. First, the breast with no pathology was imaged, then the breast with the suspected lesion. Both CranioCaudal (CC) and MedioLateral Oblique (MLO) views were collected. For each view, the CESM technique allowed for obtaining two images: an LE acquisition at 26–30 kVp and an HE acquisition at 45–49 kVp, with these values depending on breast density and thickness. Image acquisition was completed within 5 min (as shown in Figure 2), after which LE and HE images were recombined in order to suppress background and made evident the CM uptake. The dual-energy subtraction technique is less sensitive to movement artifact than traditional temporal subtraction, although motion blur may be sometimes observed on RC images due to movements between the acquisition of low- and high-energy images.

All of the images obtained were in DICOM format and were evaluated by a dedicated radiologist with more than 10 years of experience in reading mammography and breast MR images and trained in reading contrast-enhanced images.

#### 2.1.2. Inclusion and Exclusion Criteria

For this study, we have considered women referred to *I.R.C.C.S. Istituto Tumori “Giovanni Paolo II”* of Bari (Italy) from the breast cancer screening phase between March 2017 and June 2018.

Patients undergone to CESM had indications for breast MRI, but, for several reasons, they could not perform it. In more detail, due to the presence of radiation exposure in CESM, in our Institute, the use of this method was applied only as a second alternative to MRI in case of contraindications or impossibility of the patient to perform MRI. CESM was preferred to resonance even for patients who had to perform urgent MRI for therapies or programmed surgery but that have not found access to MRI, as indicated by the European guidelines on CESM [28,29]. Our observational study was approved by medical ethics committee of the Institute. All eligible patients provided a written informed consent prior to undergoing the CESM examination.

Patients were excluded when: they were assumed to be pregnant or breastfeeding; they had contraindications to CESM including a history of an anaphylactoid or anaphylactic reaction to any contrast media or impaired renal function of chronic kidney disease stage 3 and higher (e.g., creatinine clearance <60 mL/min); they had received any contrast material within 24 h prior to the contrast-enhanced spectral mammography; they had breast implants; they had already undergone surgery, hormone treatment or radiation therapy for the index lesion; they had already started neoadjuvant chemotherapy before inclusion.

#### 2.1.3. Experimental Dataset

We have selected images in MLO or CC view of 53 patients aged between 37 and 76 years (with a mean of 52.2±10.1 years), resulting as positive to CESM examination for the presence of at least one findings after histological examination. A total of 58 ROIs containing primary and, if present, also secondary lesions from 0.6 to 13.5 cm was manually identified using a bounding box and classified by a radiologist of our Institute dedicated to senologic diagnostics according to the BIRADS classification [30]: lesions belonging to BIRADS 2 and 3 classes were labeled as benign, while lesions belonging to BIRADS 4 and 5 classes were considered as malignant. Then, this radiologist classification was compared to the histological diagnosis based on bioptic sampling: as result, 24 lesions were benign (BIRADS 2) and 34 malignant (BIRADS 5). Moreover, for each patient, our radiologist identified an enhanced ROI not containing any lesions.

**Figure 3:**
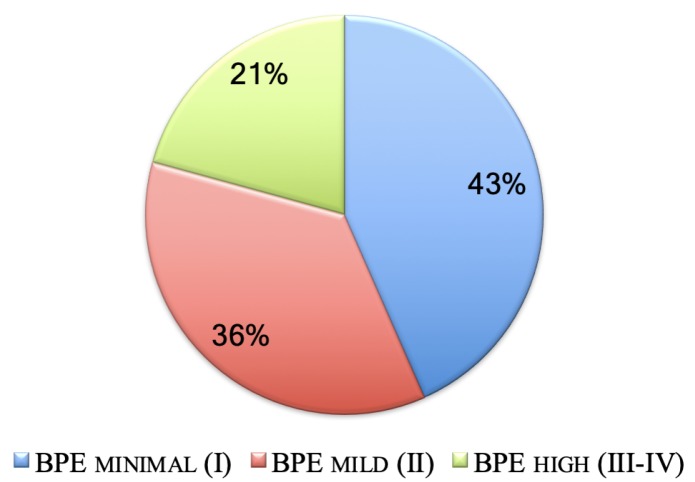
BPE distribution of the patients (%) undergone to CESM examinations and analyzed in this study.

The patient distribution in three BPE classes, i.e., minimal, mild and high (moderate or marked), is shown in Figure 3. All ROIs were extracted on both low-energy and recombined images.

### 2.2. Methods

In this paper, we presented a fully automated model to classify benign and malignant ROIs and to discriminate an ROI containing lesions from an enhanced ROI without any lesions, both extracted from CESM images.

**Figure 4:**
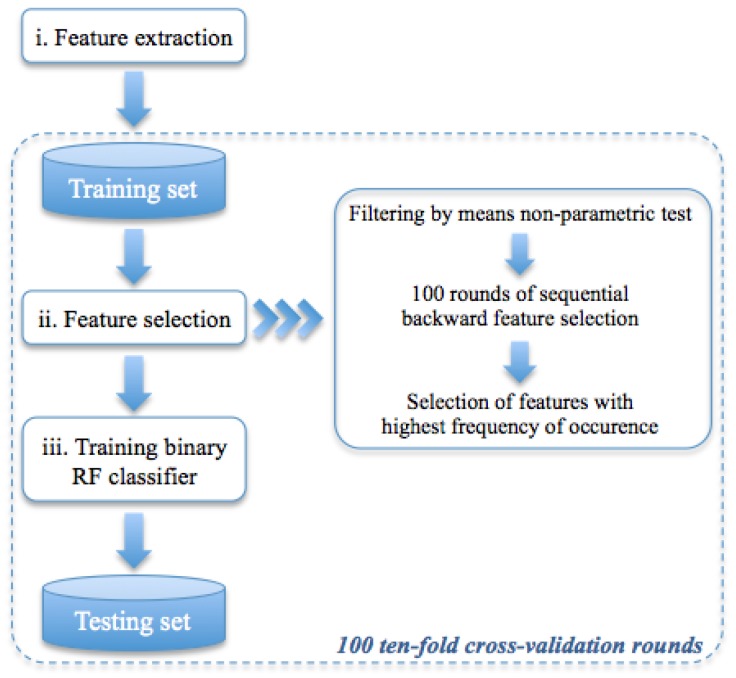
Flow-chart of the proposed model. In the first phase, a set of features on each ROI is extracted, then a sub-set of significant features is selected; finally, binary RF classifiers are trained.

As schematically illustrated in Figure 4, the method was developed in three phases: (i) for each ROI, a set of textural features was extracted, (ii) a feature sub-set was selected on training set by means 100 round of stepwise selection, and, (iii) finally, a Random Forest (RF) binary classifier [31] was trained to discriminate ROIs using the selected feature sub-set. Feature extraction, selection, analysis, and classification model generation were performed using the MATLAB R2017a (Mathworks, Inc., Natick, MA, USA) software.

#### 2.2.1. Feature Extraction

Starting from each ROI extracted from both original LE and RC images, five feature sets were extracted by using different techniques.

##### Statistical Features

From each original ROI, not pre-processed by any imaging technique, we have extracted a first set of statistical features: mean, standard deviation and their ratio, variance, skewness, entropy, relative smoothness, kurtosis, minimum and maximum values of gray-level and their difference. These 11 features were extracted from each LE and RC original ROI, forming the *STAT set* with 22 features.

##### Interest Point, Corner and Region Detection

Another set of features was defined by counting the number of interest points, corners and regions detected on each original ROIs by using five different algorithms, such as Scale Invariant Feature Transform (SIFT) [32,33], Minimum Eigenvalue (MinEigen) algorithm [34], Features from Accelerated Segment Test (FAST) algorithm [35,36], Binary Robust Invariant Scalable Keypoints (BRISK) method [37], and Maximally Stable Extremal Regions (MSER) [38]. These methods allowed for detecting significant points, corners and regions on an image, in order to describe local features by means of particular functions, depending on the problem to be solved and exploiting some invariant properties of image transformations. The feature set thus obtained and called *COUNT set*, containing a total of 10 features, had been successfully used in our previous work on digital mammographic images for the classification of clustered microcalcifications [39,40].

##### Gradient Image

We considered some statistical features (mean, variance, skewness, entropy, relative smoothness and kurtosis), extracted from the gradient’s magnitude and direction of each LE and RC original ROI. This feature set was labeled as *GRAD set* and totally formed by 24 features. As known, the gradient of a two-variable function f(x,y) is represented by a vector of the partial derivatives in the *x* and *y* directions ($f_{x}$, $f_{y}$) [41]. In the case of an image, it is represented by a discrete function of (*x*, *y*) for which the derivatives are not defined. Thus, the gradient could be calculated making some hypotheses about the intensity function of the image and assuming that there is a continuous intensity function sampled at the image points. In this way, the gradient of the image can be approximated by the convolution with a kernel, such as the Sobel or Prewitt operator, and, mathematically, its vector can be calculated at each pixel with a magnitude (*Gmag*) and a direction (*Gdir*) given by $\sqrt{f_{x}^{2}+f_{y}^{2}}$ and $\arctan(f_{y}/f_{x})$, respectively; in this work, they were computed by using a Sobel kernel as a two-dimensional method, to each pixel and its neighbours.

##### Haar Wavelet Transform

**Figure 5:**
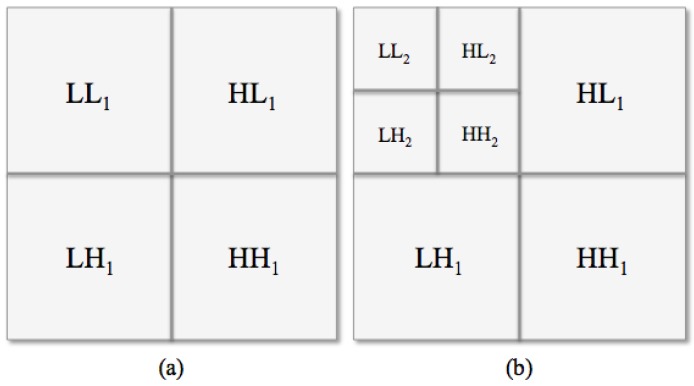
Haar decomposition schema. (**a**) one- and (**b**) two-level Haar decomposition.

By using a texture analysis approach, we exploited a wavelet transform, known as Haar wavelet [41,42], since the image texture depends on the scale at which an image is analyzed. This wavelet function allows for decomposing an image in a sequence of sub-images: the original ROI was high-pass filtered in three directions (horizontal, vertical and diagonal, Figure 5a, top right and bottom), then low-pass filtered and downscaled (Figure 5a, top left). To compute the successive levels of decomposition, the process was iterated on the downscaled sub-image (Figure 5b, top left). In particular, we considered two levels of decomposition and we extracted a set of six features (mean, variance, skewness, entropy, relative smoothness and kurtosis) from each eight sub-ROI, i.e., Low-Low (LL), High-Low (HL), Low-High (LH), and High-High (HH) for levels 1 and 2, from both LE and RC sub-images. The so called *HAAR set*, including a total of 96 features, was obtained.

##### Gray-Level Co-Occurence Matrix

**Figure 6:**
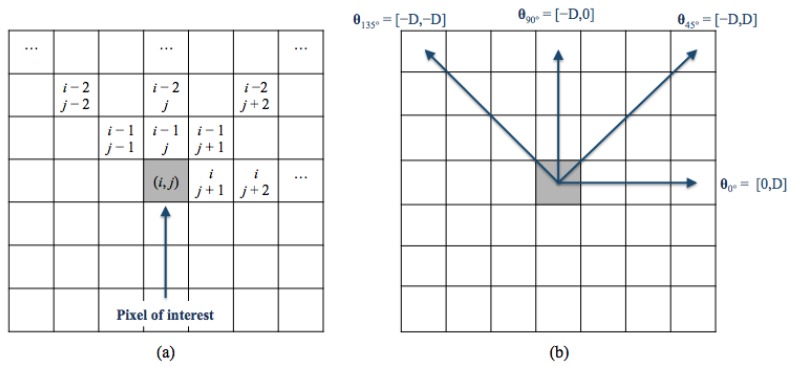
(**a**) The spatial relationships of pixels; (**b**) the GLCM directions. *D* is the offset and represents the distance between each pixels and the pixel of interest.

The Gray-Level Co-occurence Matrix (GLCM) [43,44,45] is another common technique to extract textural features. The GLCM is obtained counting how many times the gray-level intensity value occurs to another in a specific spatial relationship to each pixel (*i*, *j*) (Figure 6a). This relationship, known as *offset*, is fixed as the distance between a pixel and its neighbours with respect to a specific direction (Figure 6b). A texture function reduces the number of intensity values in gray-scale image from 256 to eight in order to determine the size of the GLCM matrix. Thus, we obtained a set of statistical features (contrast, correlation, cluster prominence, cluster shade, dissimilarity, energy, entropy, homogeneity, sum average, sum variance, sum entropy, difference entropy and normalized inverse difference moment) extracted from the co-occurrence matrix of each sub-ROI previously decomposed by the Haar transform (HL, LH and HH) only at the first level in four directions ($\theta =0^{\circ },45^{\circ },90^{\circ },135^{\circ }$), resulting in 156 features. This last set was named *GLCM set* and, considering both LE and RC sub-ROIs, it was formed by a total of 312 features.

**Figure 7:**
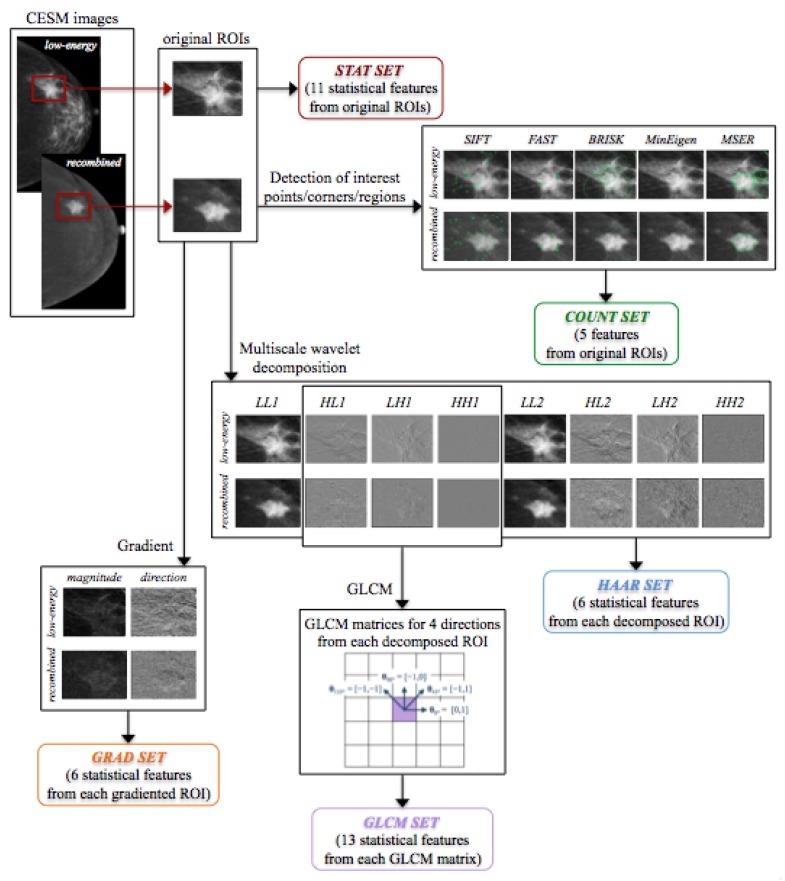
Scheme of the extraction of each feature set.

Figure 7 shows a scheme of the extraction of each feature set used in this work, starting from both original LE and RC images and their extracted ROIs.

#### 2.2.2. Classification Model

The general structure of the proposed classification model is shown in Figure 4.

We first selected only the features useful in data analysis. Specifically, the non-parametric Wilcoxon–Mann–Whitney test [46] was used to verify whether the medians of distributions of the two classes of the binary problem were equal. Then, a backward feature selection algorithm [47] combined with a Naïve Bayes classifier was adopted in order to identify the most discriminant features for the binary classification problem. The sequential backward selection algorithm identified a sub-set of features that best predicted the expected result by sequentially removing features from the initial candidate set until there was no improvement in prediction on 10-fold cross-valuation. The overall feature sets were sorted in descending order by the occurrence frequency in a final sub-set identified on 100 rounds of the sequential feature selection algorithm. Then, a state-of-the-art machine learning classifier, such as RF classifier, was trained to solve the binary discrimination problem by selecting iteratively an increasing number of the ordered features. The main advantage of RF classifier with respect to standard classification algorithms is its robustness against overfitting; moreover, it is easy to tune because it depends only on two parameters that are the number of trees to be grown and the number of features to pick at each node split. Therefore, it was a good choice for an exploratory analysis. A standard configuration of RF was adopted with 100 trees and 20 features (as described in [31]) randomly selected at each split. Other different machine learning techniques of the state-of-the-art have been evaluated, but no improvement in performance with respect to those obtained with an RF classifier was achieved.

The proposed model was evaluated on two binary discrimination problems, i.e., benign vs. malignant ROIs and normal tissue vs. parenchymal background. The performance of the prediction model was evaluated on 100 ten-fold cross-validation rounds in terms of Area Under the Curve (AUC) of the Receiver Operating Characteristic (ROC) curve and also accuracy, sensitivity and specificity (in terms of median and InterQuartile Range—IQR) calculated by identifying the optimal threshold by means of Youden’s index on the ROC curve [48]. Finally, in order to compare performance also related to unbalanced sub-samples, we provided the Matthews Correlation Coefficient (MCC) calculated as

(1) $$MCC=\frac{\left(TP\times TN)-(FP\times FN\right)}{\sqrt{\left((TP+FP)\times (TP+FN)\times (TN+FP)\times (TN+FN)\right)}},$$

where *TP*, *TN*, *FP* and *FN* were the true positive, true negative, false positive and false negative rates, respectively.

The MCC is often used in machine learning as a measurement of the quality of binary classification [49] to compare performance results when the classes are very different in sizes because it also considers both true and false positives and false negatives against other similar coefficients [50]. The MCC is a correlation coefficient between true and predicted binary classification having a real value between 0 and 1: the closer to one the value of the coefficient is, the better the classification is.

## 3. Results

### 3.1. Human-Reader Diagnostic Accuracy

Our radiologist first evaluated only LE images (similar to standard 2D digital mammograms), and then jointly LE and RC images (denoted as CESM images) providing a diagnosis based on BIRADS classification, as described in Section 2.1.3. In Table 1, we compared the diagnostic performances of our radiologist obtained by reading only LE images or CESM ones with respect to micro-histological results (24 benign and 34 malignant). Out of 53 patients, by evaluating only LE images, 57 lesions were detected, of which 38 were classified as malignant, while by evaluating also RC images, 58 lesions were identified by the radiologist, of which 38 were classified as malignant.

Compared to the gold standard provided by micro-histological results, CESM showed a high diagnostic sensitivity, but a lower specificity (100% vs. 83.3%).

Moreover, CESM diagnostic performances presented an improvement by about 11%, 8%, and 13% in accuracy, sensitivity and specificity, respectively, with respect to standard mammography ones. However, it should be stressed that the performances measured by the observation of an LE image are likely overestimated with respect to a standard mammographic image. This is due both to the best image quality and the administration of the contrast medium, which allow a contrastographic assessment of the breast highlighting the areas that capture the contrast medium, as for magnetic resonance images, typical expression of neoplastic neo-angiogenesis.

### 3.2. Prediction Accuracy of Benign and Malignant ROIs

The proposed method has been trained on a sub-set of significant features identified by means of a sequential backward feature selection algorithm. Specifically, as described in Section 2.2, a binary RF classifier was trained on a significant feature sub-set identified by the feature selection process. We showed the performance classification results for an increasing number of features sorted by their occurrence frequency defined by the sequential feature selection algorithm.

**Figure 8:**
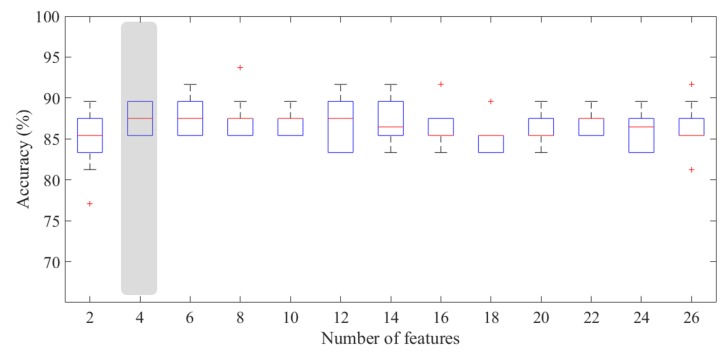
Accuracy for benign/malignant classification with respect to the number of features. The gray box highlights the accuracy peak obtained for n=4, significantly different to the model with only two features (*p*-value of Wilcoxon–Mann–Whitney test <0.05).

Since the number of benign ROIs was reduced with respect to that of malignant ones, we considered it appropriate to apply the under-sampling technique in order to solve the benign vs. malignant ROI discriminant problem. Therefore, we have trained a classifier on balanced sub-set of 48 ROIs (24 benign and 24 malignant) by removing randomly instances from the over-represented class to reduce the training bias. As highlighted in Figure 8, the experimental outcomes calculated on 100 rounds of ten-fold cross-validation showed that the developed model was highly performing even using a reduced number of features. Indeed, the proposed model showed a median AUC value of 93.1% (IQR of 90.5–93.4%) with only four features; selecting an optimal threshold by means of Youden’s test at each round, the model reached a median accuracy of 87.5% (IQR of 85.4–89.6%), a median sensitivity of 87.5% (IQR of 83.3–91.7%), and a specificity of 91.7% (IQR of 87.5–91.7%). Moreover, we have calculated the performance for each BPE class. As shown in Table 2, the model was highly performing in each of the three classes, i.e., minimal, mild and high (moderate or marked), with a median accuracy value of 86.0% (IQR of 84.0–88.0%), 95.5% (IQR of 90.0–100%), and 83.3% (IQR of 81.3–84.3%), respectively. However, the three classes are not numerically balanced, making the standard measurements of performance not comparable; therefore, we evaluated the goodness of the classification performance by calculating the MCC. The MCC for the three classes was 0.84, 0.91, and 0.68, respectively, confirming that, even when the background parenchymal enhancement was high, the classification was still moderately performing.

As summarized in Table 3, our classification model showed an increase of diagnosis performance by over 8% in detecting benign lesions (i.e., specificity) with respect to the human reader performances. On the contrary, the radiologist exceeded the proposed model in detecting malignant lesions.

### 3.3. Prediction Accuracy of Normal and Abnormal ROIs Characterized by Mild/High BPE

Same classification model has been applied to the recognition problem of normal and abnormal regions when the patient’s breast was characterized by a mild or high BPE. Therefore, we have trained a classifier on a sub-set of ROIs (31 containing lesions and 32 without any lesions, that were enhanced ROIs).

The experimental results, intended as the first approach to the development of an automated detection system, have shown that at least 12 features were required to obtain performing results: a greater number of features did not seem to record an appreciable increase in accuracy. Indeed, with only 12 features, the model reached a median AUC value of 85.0% (IQR of 84.5–87.4%) and a median accuracy of 82.5% (IQR of 79.0–82.5%), calculated by selecting an optimal threshold using Youden’s test. Nevertheless, in terms of recognition of regions characterized by a lesion, the performances were not particularly appreciable (median sensitivity of 70.3% with an IQR of 68.8–84.4%), compared to the recognition of a normal region (median specificity of 94.0% with an IQR of 88.0–96.0%). This result seems to depend on the BPE degree, since, as shown in Table 4, the performances were better when BPE was mild (median value of MCC equals to 0.71).

## 4. Discussion

CESM is a mammographic technique based on a dual-energy exposure after a single injection of an iodinated contrast medium whose LE image yielded as part of a CESM exam can be overlapped on a digital mammography image according to modern quality standards.

In the early studies published in the literature, CESM had a higher sensitivity than standard mammography in the detection of breast lesions [51,52]. In these works, it was shown how the use of CESM in addition to digital mammography significantly improved the performance of the human reader compared to those obtained with the use of mammography alone.

According to literature [53,54,55], our experience showed that the performances of human reader by CESM instrumentation get a low specificity, probably due to the hormonal influence of the patients which generates an inevitable variation of BPE in terms of both diffusion and intensity. Moreover, CESM diagnostic performances presented an improvement by about 13% in accuracy, and in particular over 8% in specificity with respect to standard mammography ones.

Nevertheless, this estimate seems to reflect what was made evident by recent studies, i.e., the greatest increase observed in terms of specificity. Indeed, in [56], it was reported that, by using CESM instrumentation, the cancer diagnosis improved by 15%, 9%, and 26% in accuracy, sensitivity and specificity, respectively, compared to digital mammography; however, in [51], the authors showed that the use of CESM improved the cancer diagnosis by 33.6%, 3.1%, and 45.7%.

CESM shows interesting results in terms of diagnostic sensitivity, compatible with those obtained by MRI: in [57], the sensitivity for both techniques was 100%, while, in [14], it was 100% by CESM and 93% by breast MRI. Then, CESM can be considered a valid alternative to MRI in the case of contraindications to the latter [28]. On the basis of state-of-the-art comparative results, CESM also has better tolerance and less discomfort compared to MRI, as shown in [51,58,59]. Nevertheless, both CESM and MRI present false positive cases, particularly in some fibroadenomas [10]. Moreover, the diagnosis by CESM can still be considered operator-dependent, also due to the current lack of objective measurement system of pathological enhancement (I/T curves, ROI) [10,52,53,60].

In this work, we proposed a CADx system as a support tool to human reader aimed at reducing unnecessary biopsies and later surgeries. The experimental results have pointed out that the benign/malignant problem can be effectively solved with a number of features decidedly contained (no more than 4), as shown in Figure 8, achieving a median AUC value of 93.1% and an accuracy of 87.5%. Specifically, the best performances were obtained by using the four most frequently selected features highlighted in Table 5, for which the accuracy peak is reached. For this problem, the most discriminating features were the number of interest points obtained by the SIFT method and the variance calculated on a Haar decomposition (LL2); in addition, the relative smoothness measurement, calculated on the original RC ROI and the gradient magnitude of its corresponding LE ROI, had significant information content discriminating benign/malignant ROIs. It is worth noting that the four most significant features belong to four of the five categories of features, confirming the importance of the multivariate feature selection analysis carried out in this work. In Table 5, all features used for benign/malignant ROI classification with a frequency significantly different from the chance are also summarized.

**Figure 9:**
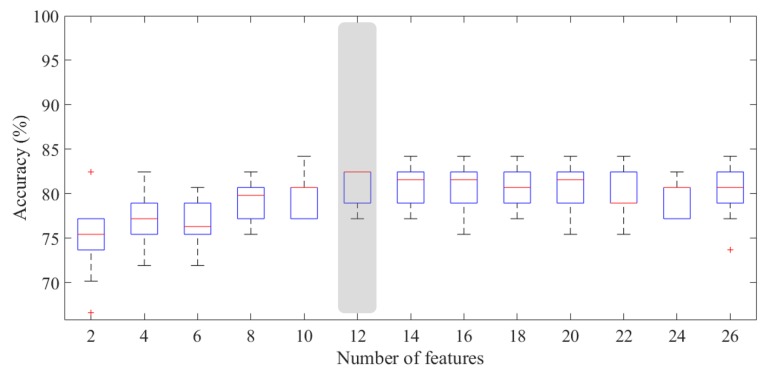
Accuracy for background/lesion classification with respect to the number of features. The gray box highlights the accuracy obtained for n=12, significantly different to the model with only two features (*p*-value of Wilcoxon–Mann–Whitney test <0.05).

With respect to the ROI classification into background/lesion, all features with a frequency significantly different from the chance are listed in Table 6. This is a much more complex problem that currently does not present references in literature. For this case, the 12 most frequently selected features for which the accuracy peak (82.5%, corresponding to a median AUC value of 85.0%) is reached are highlighted in Figure 9. This excellent result was obtained by using mainly features calculated on various Haar decompositions (LH1, LH2 and LL2), and GLCM matrices on LH1 Haar decomposition with different directions. Moreover, we can note that most features were extracted from RC images, where a suspicious lesion is not generally masked by the denser breast parenchyma, as instead happens in LE images. The prevalence of RC-derived features could therefore emphasize the importance of recombined images that characterize CESM.

The diagnostic problem can be solved effectively with a limited number of textural features (no more than 4), achieving high performances (sensitivity and specificity of 87.5% and 91.7%) comparable to the state-of-the-art, although this comparison is purely qualitative because all models are evaluated on private databases, and so their results could not be repeated. The literature is not really particularly nourished with references to the development of expert systems supporting the analysis of CESM images. However, there are two relevant works proposing automated predictive models. In [24], an ROI classification model was performed by training an SVM classifier on a textural and morphological feature set extracted from 50 lesions (24 malignant, 26 benign) segmented by the radiologists, and reaching a sensitivity and a specificity of 88% and 92%, respectively. With respect to this work, our model does not need a manual segmentation of lesions by radiologist but only an identification of suspicious areas as human operation, and it can be able to characterize the lesions by a reduced number of features.

In [26], a sensitivity of 100% and a specificity of 66% was achieved by incorporating BIRADS textual features provided by the radiologists as an additional input to Convolutional Neural Network (CNN). In this work, the authors used only textual descriptors provided by the radiologist and combined with CESM pixel information extracted directly from the 129 images containing lesions of various nature (56 malignant, 73 benign). On the contrary, our machine learning model is supported by an important radiomic analysis for the characterization of benign and malignant ROIs, useful for defining potential diagnostic biomarkers of the disease.

Moreover, in these works, no reference is made to diagnostic performances for each BPE degree: this is a very important factor in the evaluation of the results achieved with the CADx because, as already mentioned, the value of BPE degree could affect the distinction between a lesion and the non-enhanced background.

## 5. Conclusions

Recent feasibility studies suggest that CESM is a useful investigation tool, and it can provide pre-operative staging and accurate treatment planning in breast cancer patients with an accuracy not less than MRI [9]. The intrinsic characteristics of this method make its use effective, especially in cases in which MRI examinations can not perform or have some limitations (absolute or relative contraindications, pre-menopausal women with high BPE). However, the diagnosis by CESM can still be considered very subjective and dependent on the operator experience.

In this context, we proposed an automated expert system for discriminating benign and malignant ROIs. Our experimental results showed that it could offer radiologists a reliable support diagnostic tool. Indeed, with respect to the human reader performances, our classification model showed an increase of diagnosis performance by over 8% in terms of specificity, reducing the false positives rate and thus the problems related to over-diagnosis, such as unnecessary biopsies and later surgeries.

Moreover, the proposed model is completely automated and does not require a lesion segmentation or indications about textual descriptors by radiologists, but only the identification of a suspicious area. Using only morphological features, experimental results are promising with respect to works at the state-of-the-art also when BPE is moderate or marked.

Nevertheless, in this work, we have not considered the effects of the patient hormonal state during the radiological examinations, which can generate a variation of BPE in terms of both diffusion and intensity. Indeed, in young women, intense hormonal activity is almost always associated with a high BPE, but a moderate-high BPE can also be present in menopausal women, although in certainly smaller numbers. Therefore, in the next stage of our studies, it will be necessary to investigate these effects on diagnostic performances of CESM instrumentation.

## Figures and Tables

**Table 1:** Diagnostic performances of human reader on only LE and CESM images with respect to micro-histological investigation. We denote with “CESM images” the joint reading of LE and RC images.

Diagnostic Test Parameter	Only LE Images	CESM Images	Micro-Histological Results (Only LE/CESM)
No. of selected patients	53	53	53/53
No. of selected breasts	47	48	47/48
No. of selected lesions	57	58	57/58
No. of malignant lesions (TP)	38 (34)	38 (34)	34/34
No. of benign lesions (TN)	16 (16)	20 (20)	23/24
Sensitivity [CI 95%]	91.2% (88.8–93.5%)	100% (95.0–100%)	
Specificity [CI 95%]	69.6% (67.9–71.2%)	83.3% (81.2–85.4%)	
Accuracy [CI 95%]	82.5% (80.4–84.6%)	93.1% (90.7–95.5%)	
MCC	0.63	0.86	
Size of lesions: mean ± SD (mm)	26.1±17.9	28.8±18.3	
Size of the smallest lesion detected (mm)	6	6	

**Table 2:** ROI classification into benign and malignant, in terms of accuracy, sensitivity, specificity, and MCC with respect to BPE degree. For each BPE class, the number of benign/malignant (B/M) ROIs is shown.

	BPE I (15B/10M)	BPE II (4B/7M)	BPE III-IV (5B/7M)	Overall Dataset (24B/24M)
Accuracy (%)	86.0 (84.0–88.0)	95.5 (90.9–100)	83.3 (81.3–84.3)	87.5 (85.4–89.6)
Sensitivity (%)	75.0 (70.0–80.0)	100 (100–100)	92.9 (85.7–100)	87.5 (83.3–91.7)
Specificity (%)	96.7 (86.7–100)	100 (75.0–100)	80.0 (60.0–80.0)	91.7 (87.5–91.7)
MCC	0.84 (0.72–0.91)	0.91 (0.81–1)	0.68 (0.66–0.71)	0.76 (0.74–0.79)

**Table 3:** Result comparison between human reader and proposed model.

	Human Reader (24B/34M)	Proposed Model (24B/24M)
Accuracy (%)	93.1	87.5
Sensitivity (%)	100	87.5
Specificity (%)	83.3	91.7
MCC	0.86	0.76

**Table 4:** ROI classification into background and lesion, in terms of accuracy, sensitivity and specificity with respect to BPE degrees. The minimal BPE class (I) is not included.

	BPE II (15 Normal/20 Abnormal)	BPE III–IV (10 Normal/12 Abnormal)	Overall Dataset
Accuracy (%)	82.9 (80.0–88.6)	77.3 (77.3–81.8)	82.5 (79.0–82.5)
Sensitivity (%)	70.0 (65.0–80.0)	75.0 (66.7–91.7)	70.3 (68.8–84.4)
Specificity (%)	100 (100–100)	85.0 (85.0–90.0)	94.0 (88.0–96.0)
MCC	0.71 (0.67–0.79)	0.57 (0.55–0.65)	0.65 (0.63–0.69)

**Table 5:** All features whose selection frequency is significantly different from the chance in the benign/malignant classification (*p*-value null model test <0.05). Direction and level of the Haar decomposition, GLCM direction, and gradient’s magnitude or direction of the extracted features are shown.

Feature Set	Feature	ROI Type	Frequency (%)
COUNT	SIFT	LE	84.8
HAAR	Variance_LL2	RC	50.4
STAT	RelativeSmoothness	RC	45.8
GRAD	RelativeSmoothness_Gmag	LE	43.8
HAAR	Variance_LL1	RC	34.8
STAT	Variance	RC	34.2
GRAD	Variance_Gmag	LE	31.9
GLCM	ClusterProminence_HL1 ($\theta =90^{\circ }$)	RC	31.0
GLCM	Correlation_LH1 ($\theta =0^{\circ }$)	RC	30.3
HAAR	Variance_LH1	LE	29.9
HAAR	RelativeSmoothness_HL2	RC	29.5
HAAR	Variance_LH2	LE	23.6
GLCM	Homogeneity_LH1 ($\theta =0^{\circ }$)	RC	22.4
STAT	Standard Deviation	RC	20.3
HAAR	Variance_HL2	RC	19.5
STAT	Maximum − Minimum	RC	17.8
COUNT	MinEigen	RC	16.4

**Table 6:** All features whose selection frequency is significantly different from the chance in the background/lesion classification (*p*-value null model test <0.05). Direction and level of the Haar decomposition, and GLCM direction of the extracted features are shown.

Feature Set	Feature	ROI Type	Frequency (%)
HAAR	Mean_LL2	RC	41.0
GLCM	SumEntropy_LH1 ($\theta =90^{\circ }$)	RC	40.6
GLCM	Entropy_LH1 ($\theta =135^{\circ }$)	RC	25.4
HAAR	Mean_LL2	LE	23.8
HAAR	Variance_LH1	RC	23.6
GLCM	Entropy_LH1 ($\theta =90^{\circ }$)	RC	22.5
HAAR	Entropy_LH1	RC	22.3
GLCM	SumEntropy_LH1 ($\theta =45^{\circ }$)	RC	21.2
HAAR	RelativeSmoothness_LH1	RC	18.4
HAAR	RelativeSmoothness_LH2	RC	17.8
HAAR	Variance_LH2	RC	17.7
GLCM	Energy_LH1 ($\theta =135^{\circ }$)	RC	17.5
HAAR	Mean_LL1	RC	17.4
HAAR	Variance_LL2	RC	17.4
HAAR	Mean_LL1	LE	17.2
GLCM	Entropy_LH1 ($\theta =0^{\circ }$)	RC	14.6
STAT	Minimum	RC	14.5
GLCM	ClusterProminence_HH1 ($\theta =0^{\circ }$)	RC	14.3
COUNT	MSER	RC	13.5
HAAR	Variance_LL1	RC	13.2
HAAR	RelativeSmoothness_LL1	RC	12.6
GLCM	Entropy_LH1 ($\theta =45^{\circ }$)	RC	12.5

## Abbreviations

The following abbreviations are used in this manuscript:

ADC	Apparent Diffusion Coefficient
AUC	Area Under the Curve
BPE	Background Parenchymal Enhancement
BRISK	Binary Robust Invariant Scalable Keypoints
CADx	Computer Automated Diagnosis
CC	CranioCaudal
CESM	Contrast-Enhanced Spectral Mammography
CI	Confidence Interval
CM	Contrast Medium
CT	Computerized Tomography
DWI	Diffusion-Weighted Imaging
FAST	Features from Accelerated Segment Test
FFDM	Full-Field Digital Mammography
FN	False Negative
FP	False Positive
Gdir	Gradient direction
Gmag	Gradient magnitude
GLCM	Gray-Level Co-occurence Matrix
HE	High-Energy
HH	High-High
HL	High-Low
IQR	InterQuartile Range
LE	Low-Energy
LH	Low-High
LL	Low-Low
MinEigen	Minimum Eigenvalue
MCC	Matthews Correlation Coefficient
MLO	MedioLateral Oblique
MRI	Magnetic Resonance Imaging
MSER	Maximally Stable Extremal Regions
RC	ReCombined
RF	Random Forest
ROC	Receiver Operating Characteristic
ROI	Region Of Interest
SD	Standard Deviation
SIFT	Scale Invariant Feature Transform
SVM	Support Vector Machine
TN	True Negative
TP	True Positive

## References

[1] Bray F., Ferlay J., Soerjomataram I., Siegel R.L., Torre L.A., Jemal A. (2018). Global cancer statistics 2018: GLOBOCAN estimates of incidence and mortality worldwide for 36 cancers in 185 countries. CA Cancer J. Clin..

[2] Cronin K.A., Lake A.J., Scott S., Sherman R.L., Noone A.M., Howlader N., Henley S.J., Anderson R.N., Firth A.U., Ma J. (2018). Annual Report to the Nation on the Status of Cancer, part I: National cancer statistics. Cancer.

[3] Cerello P., Bagnasco S., Bottigli U., Cheran S.C., Delogu P., Fantacci M.E., Fauci F., Forni G., Lauria A., Torres E.L. (2005). GPCALMA: A Grid-based tool for mammographic screening. Methods Inf. Med..

[4] Fauci F., Raso G., Magro R., Forni G., Lauria A., Bagnasco S., Cerello P., Cheran S.C., Torres E.L., Bellotti R. (2005). A massive lesion detection algorithm in mammography. Phys. Med..

[5] Cheung Y.C., Lin Y.C., Wan Y.L., Yeow K.M., Huang P.C., Lo Y.F., Tsai H.P., Ueng S.H., Chang C.J. (2014). Diagnostic performance of dual-energy contrast-enhanced subtracted mammography in dense breasts compared to mammography alone: Interobserver blind-reading analysis. Eur. Radiol..

[6] Vestito A., Lorusso V., Faggian A., Gaballo A., Garasto E., Mangieri F.F., La Forgia D., Ancona A. (2014). Contrast Enhanced Spectral Mammography: la nostra esperienza. Il Giornale Italiano di Radiol. Med..

[7] Masala G., Tangaro S., Golosio B., Oliva P., Stumbo S., Bellotti R., De Carlo F., Gargano G., Cascio D., Fauci F. (2007). Comparative study of feature classification methods for mass lesion recognition in digitized mammograms. Image.

[8] Tagliafico A.S., Mariscotti G., Valdora F., Durando M., Nori J., La Forgia D., Rosenberg I., Caumo F., Gandolfo N., Sormani M.P. (2018). A prospective comparative trial of adjunct screening with tomosynthesis or ultrasound in women with mammography-negative dense breasts (ASTOUND-2). Eur. J. Cancer.

[9] Fallenberg E., Dromain C., Diekmann F., Engelken F., Krohn M., Singh J., Ingold-Heppner B., Winzer K., Bick U., Renz D.M. (2014). Contrast-enhanced spectral mammography versus MRI: Initial results in the detection of breast cancer and assessment of tumour size. Eur. Radiol..

[10] Patel B.K., Lobbes M., Lewin J. (2018). Contrast enhanced spectral mammography: A review. Seminars in Ultrasound, CT and MRI.

[11] Lalji U., Jeukens C., Houben I., Nelemans P., van Engen R., van Wylick E., Beets-Tan R., Wildberger J., Paulis L., Lobbes M. (2015). Evaluation of low-energy contrast-enhanced spectral mammography images by comparing them to full-field digital mammography using EUREF image quality criteria. Eur. Radiol..

[12] Fallenberg E.M., Dromain C., Diekmann F., Renz D.M., Amer H., Ingold-Heppner B., Neumann A.U., Winzer K.J., Bick U., Hamm B. (2014). Contrast-enhanced spectral mammography: Does mammography provide additional clinical benefits or can some radiation exposure be avoided?. Breast Cancer Res. Treat..

[13] James J., Tennant S. (2018). Contrast-enhanced spectral mammography (CESM). Clin. Radiol..

[14] Łuczyńska E., Heinze-Paluchowska S., Hendrick E., Dyczek S., Ryś J., Herman K., Blecharz P., Jakubowicz J. (2015). Comparison between breast MRI and contrast-enhanced spectral mammography. Med. Sci. Monit. Int. Med. J. Exp. Clin. Res..

[15] Prionas N.D., Lindfors K.K., Ray S., Huang S.Y., Beckett L.A., Monsky W.L., Boone J.M. (2010). Contrast-enhanced dedicated breast CT: Initial clinical experience. Radiology.

[16] Onega T., Tosteson A.N., Weiss J., Alford-Teaster J., Hubbard R.A., Henderson L.M., Kerlikowske K., Goodrich M.E., O’Donoghue C., Wernli K.J. (2016). Costs of diagnostic and preoperative workup with and without breast MRI in older women with a breast cancer diagnosis. BMC Health Serv. Res..

[17] Sankatsing V.D., Heijnsdijk E.A., van Luijt P.A., van Ravesteyn N.T., Fracheboud J., de Koning H.J. (2015). Cost-effectiveness of digital mammography screening before the age of 50 in T he N etherlands. Int. J. Cancer.

[18] Gocgun Y., Banjevic D., Taghipour S., Montgomery N., Harvey B., Jardine A., Miller A. (2015). Cost-effectiveness of breast cancer screening policies using simulation. Breast.

[19] Losurdo L., Basile T.M.A., Fanizzi A., Bellotti R., Bottigli U., Carbonara R., Dentamaro R., Diacono D., Didonna V., Lombardi A. (2018). A Gradient-Based Approach for Breast DCE-MRI Analysis. BioMed Res. Int..

[20] Morris E.A., Comstock C.E., Lee C.H., Lehman C.D., Ikeda D.M., Newstead G.M. (2013). ACR BI-RADS Magnetic Resonance Imaging. ACR BI-RADS, Breast Imaging Reporting and Data System.

[21] Savaridas S., Taylor D., Gunawardana D., Phillips M. (2017). Could parenchymal enhancement on contrast-enhanced spectral mammography (CESM) represent a new breast cancer risk factor? Correlation with known radiology risk factors. Clin. Radiol..

[22] King V., Brooks J.D., Bernstein J.L., Reiner A.S., Pike M.C., Morris E.A. (2011). Background parenchymal enhancement at breast MR imaging and breast cancer risk. Radiology.

[23] Sogani J., Morris E.A., Kaplan J.B., D’Alessio D., Goldman D., Moskowitz C.S., Jochelson M.S. (2016). Comparison of background parenchymal enhancement at contrast-enhanced spectral mammography and breast MR imaging. Radiology.

[24] Patel B.K., Ranjbar S., Wu T., Pockaj B.A., Li J., Zhang N., Lobbes M., Zhang B., Mitchell J.R. (2018). Computer-aided diagnosis of contrast-enhanced spectral mammography: A feasibility study. Eur. J. Radiol..

[25] Steinwart I., Christmann A. (2008). Support Vector Machines.

[26] Perek S., Kiryati N., Zimmerman-Moreno G., Sklair-Levy M., Konen E., Mayer A. (2018). Classification of contrast-enhanced spectral mammography (CESM) images. Int. J. Comput. Assist. Radiol. Surg..

[27] Lobbes M.B., Lalji U.C., Nelemans P.J., Houben I., Smidt M.L., Heuts E., De Vries B., Wildberger J.E., Beets-Tan R.G. (2015). The quality of tumor size assessment by contrast-enhanced spectral mammography and the benefit of additional breast MRI. J. Cancer.

[28] Sardanelli F., Boetes C., Borisch B., Decker T., Federico M., Gilbert F.J., Helbich T., Heywang-Köbrunner S.H., Kaiser W.A., Kerin M.J. (2010). Magnetic resonance imaging of the breast: Recommendations from the EUSOMA working group. Eur. J. Cancer.

[29] Sardanelli F., Fallenberg E.M., Clauser P., Trimboli R.M., Camps-Herrero J., Helbich T.H., Forrai G. (2017). Mammography: An update of the EUSOBI recommendations on information for women. Insights Imaging.

[30] D’Orsi C., Sickles E., Mendelson E., Morris E. (2014). 2013 ACR BI-RADS Atlas: Breast Imaging Reporting and Data System.

[31] Breiman L. (2001). Random forests. Mach. Learn..

[32] Lowe D.G. (1999). Object recognition from local scale-invariant features. Proceedings of the Seventh IEEE International Conference on Computer Vision.

[33] Lindeberg T. (2012). Scale invariant feature transform. Scholarpedia.

[34] Shi J., Tomasi C. Good features to track. Proceedings of the Ninth IEEE Conference on Computer Vision and Pattern Recognition.

[35] Rosten E., Drummond T. Fusing points and lines for high performance tracking. Proceedings of the Tenth IEEE International Conference on Computer Vision.

[36] Rosten E., Drummond T. (2006). Machine learning for high-speed corner detection. Proceedings of the 9th European conference on Computer Vision.

[37] Leutenegger S., Chli M., Siegwart R.Y. BRISK: Binary robust invariant scalable keypoints. Proceedings of the 2011 IEEE International Conference on Computer Vision (ICCV).

[38] Matas J., Chum O., Urban M., Pajdla T. (2004). Robust wide-baseline stereo from maximally stable extremal regions. Image Vis. Comput..

[39] Losurdo L., Fanizzi A., Basile T.M., Bellotti R., Bottigli U., Dentamaro R., Didonna V., Fausto A., Massafra R., Monaco A. (2018). Combined Approach of Multiscale Texture Analysis and Interest Point/Corner Detectors for Microcalcifications Diagnosis. International Conference on Bioinformatics and Biomedical Engineering.

[40] Tagliafico A.S., Valdora F., Mariscotti G., Durando M., Nori J., La Forgia D., Rosenberg I., Caumo F., Gandolfo N., Houssami N. (2018). An exploratory radiomics analysis on digital breast tomosynthesis in women with mammographically negative dense breasts. Breast.

[41] Gonzalez R.C., Woods R.E., Gonzalez R.C., Woods R.E. (2007). Image processing. Digital Image Processing.

[42] Mallat S.G. (1989). A theory for multiresolution signal decomposition: The wavelet representation. IEEE Trans. Pattern Anal. Mach. Intell..

[43] Haralick R.M., Shanmugam K. (1973). Textural features for image classification. IEEE Trans. Syst. Man Cybern..

[44] Pathak B., Barooah D. (2013). Texture analysis based on the gray-level co-occurrence matrix considering possible orientations. Int. J. Adv. Res. Electr. Electron. Instrum. Eng..

[45] Mohanaiah P., Sathyanarayana P., GuruKumar L. (2013). Image texture feature extraction using GLCM approach. Int. J. Sci. Res. Publ..

[46] Mann H.B., Whitney D.R. (1947). On a test of whether one of two random variables is stochastically larger than the other. Ann. Math. Stat..

[47] Aha D.W., Bankert R.L. (1996). A comparative evaluation of sequential feature selection algorithms. Learning from Data.

[48] Youden W. (1950). Index for rating diagnostic tests. Cancer.

[49] Matthews B.W. (1975). Comparison of the predicted and observed secondary structure of T4 phage lysozyme. Biochim. Biophys. Acta Protein Struct..

[50] Boughorbel S., Jarray F., El-Anbari M. (2017). Optimal classifier for imbalanced data using Matthews Correlation Coefficient metric. PLoS ONE.

[51] Lobbes M.B., Lalji U., Houwers J., Nijssen E.C., Nelemans P.J., van Roozendaal L., Smidt M.L., Heuts E., Wildberger J.E. (2014). Contrast-enhanced spectral mammography in patients referred from the breast cancer screening programme. Eur. Radiol..

[52] Lalji U., Houben I., Prevos R., Gommers S., van Goethem M., Vanwetswinkel S., Pijnappel R., Steeman R., Frotscher C., Mok W. (2016). Contrast-enhanced spectral mammography in recalls from the Dutch breast cancer screening program: Validation of results in a large multireader, multicase study. Eur. Radiol..

[53] Jochelson M., Lobbes M.B., Bernard-Davila B. (2017). Reply to Tagliafico AS, Bignotti B, Rossi F, et al.. Breast.

[54] Tagliafico A.S., Bignotti B., Rossi F., Signori A., Sormani M.P., Valdora F., Calabrese M., Houssami N. (2016). Diagnostic performance of contrast-enhanced spectral mammography: Systematic review and meta-analysis. Breast.

[55] Zhu X., Huang J.M., Zhang K., Xia L.J., Feng L., Yang P., Zhang M.Y., Xiao W., Lin H.X., Yu Y.H. (2018). Diagnostic value of Contrast-enhanced Spectral Mammography for screening Breast Cancer: A Systematic Review and Meta analysis. Clin. Breast Cancer.

[56] Luczyńska E., Heinze-Paluchowska S., Dyczek S., Blecharz P., Rys J., Reinfuss M. (2014). Contrast-enhanced spectral mammography: Comparison with conventional mammography and histopathology in 152 women. Korean J. Radiol..

[57] Li L., Roth R., Germaine P., Ren S., Lee M., Hunter K., Tinney E., Liao L. (2017). Contrast-enhanced spectral mammography (CESM) versus breast magnetic resonance imaging (MRI): A retrospective comparison in 66 breast lesions. Diagn. Interv. Imaging.

[58] Hobbs M.M., Taylor D.B., Buzynski S., Peake R.E. (2015). Contrast-enhanced spectral mammography (CESM) and contrast enhanced MRI (CEMRI): Patient preferences and tolerance. J. Med. Imaging Radiat. Oncol..

[59] Phillips J., Miller M.M., Mehta T.S., Fein-Zachary V., Nathanson A., Hori W., Monahan-Earley R., Slanetz P.J. (2017). Contrast-enhanced spectral mammography (CESM) versus MRI in the high-risk screening setting: Patient preferences and attitudes. Clin. Imaging.

[60] Houben I., Van de Voorde P., Jeukens C., Wildberger J., Kooreman L., Smidt M., Lobbes M. (2017). Contrast-enhanced spectral mammography as work-up tool in patients recalled from breast cancer screening has low risks and might hold clinical benefits. Eur. J. Radiol..

